# Acute torsion of the gallbladder: a case report

**DOI:** 10.1186/1757-1626-2-6641

**Published:** 2009-04-29

**Authors:** Kenan Caliskan, Alper Parlakgumus, Zafer Koc, Tarık Zafer Nursal

**Affiliations:** 1 Başkent University Faculty of Medicine, Department of General Surgery, Başkent Hastanesi Dadaloğlu Mah, 39, Sokak No: 6 Yüreğir 01250, Adana-Turkey; 2 Başkent University Faculty of Medicine, Department of Radiology Başkent Hastanesi Dadaloğlu Mah, 39, Sokak No: 6 Yüreğir 01250, Adana-Turkey

## Abstract

**Introduction:**

Torsion of gallbladder is an uncommon cause of acute abdomen. Volvulus occurs along the long axis of the gallbladder mesentery, and according to the degree of rotation symptoms, and signs may vary.

**Case presentation:**

A 79-year-old woman presented with a one-day history of acute onset of right upper abdominal pain. The patient underwent laparotomy with a preoperative diagnosis of acalculous cholecystitis with possible gangrene. At laparotomy, the gallbladder was distended, and multiple necrotic areas were observed. It was rotated more than 180 degrees clockwise around the mesentery, and cholecystectomy was performed.

**Conclusion:**

Cholecystectomy is the treatment of gallbladder torsion. Clinical signs and radiographic studies may be helpful to diagnose gallbladder torsion. Early diagnosis and surgical treatment lower the mortality of disease.

## Introduction

Torsion of the gallbladder is an uncomon condition, which can cause acute surgical abdomen. It was first described by Wendell in 1898. Since then approximately 300 cases have been reported in the literature [1]. Gallbladder volvulus may occur in any age group, however the incidence is higher in elderly women. Clinical symptoms depend on the severity of the volvulus so that, preoperative diagnosis is always difficult [2]. We report a case of necrotizing torsion of gallbladder and discuss the diagnostic, therapeutic implications, and outcomes of this entity in the context of the available literature.

## Case Presentation

A 79-year-old woman presented with a one-day history of acute onset of right upper abdominal pain. Nausea, and vomiting were also present. The vital signs were normal. Examination revealed a very tender mass in the right upper quadrant of abdomen. Laboratory investigations, including complete blood cell count, and liver function tests were within normal limits.

Abdominal ultrasound (US) examination showed a distended gallbladder, thickened gallbladder wall and fluid collection around the gallbladder. Ultrasonographic Murphy's sign was positive.

**Figure 1:**
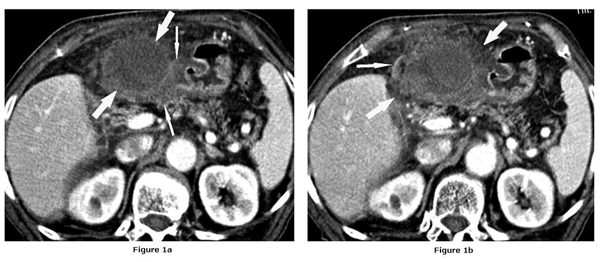
**Contrast enhanced computed tomographic images show; loss of integrity of gallbladder wall (arrows)**. Diffuse thickening of the gastric antral wall (thin arrows). **(b)** CT image obtained upper level showed fluid collection (arrows) and well-enhanced curvilinear structure on the right side of the GB indicating cystic duct located on the right side of the gallbladder (thin arrow).

No stones were detected by US. Computed tomography (CT) of the abdomen showed that the gallbladder was not within the normal anatomical liver fossa. The wall was thickened, and at one point the integrity of the wall was lost. Fluid collection all around the gallbladder indicated a "floating gallbladder" and well-enhanced curvilinear structure on the right side of the gallbladder indicating cystic duct were also identified. Diffuse edematous smooth thickening of the gastric antral wall was observed which was compatible with secondary inflammation (Figure 1a and 1b). There were no calculi in biliary system.

**Figure 2:**
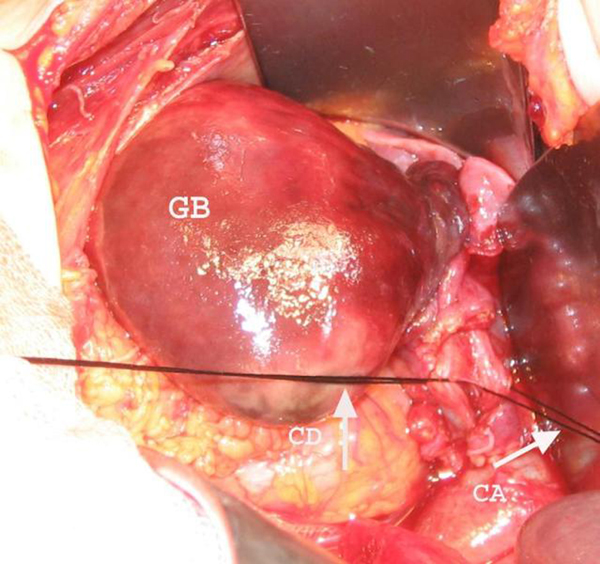
**The gallbladder was rotated more than 180 degrees clockwise around its mesentery**. GB: gallbladder, CD: cystic duct, CA: cystic artery.

The patient underwent laparotomy with a preoperative diagnosis of acalculous cholecystitis with possible gangrene. At laparotomy, the gallbladder was distended, and multiple necrotic areas were observed. It was rotated more than 180 degrees clockwise around its mesentery, and there were no gallstone in gallbladder (Figure 2). Cholecystectomy was performed in standard fashion. Histopatology of gallbladder was reported as hemorhagic transmural necrosis. The patient was discharged on second postoperative day.

## Discussion

Acute torsion of the gallbladder is an uncommon condition, characterized by rotation of the gallbladder on its mesentery along the axis of the cystic duct and cystic artery. It is more frequent in elderly patients, The female-to-male ratio is reported to be 3:1 in adults and 1:4 in the pediatric age group [2,3].

The etiology of gallbladder torsion is not well known however, the long gallbladder mesentery, and minimal attachment of the gallbladder to the liver facilitate rotation. The loss of visceral fat, and elasticity, liver atrophy, spinal deformities and weigth loss are predisposing factors that accelerate volvulus of gallbladder [3,4]. In this setting, arteriosclerotic cystic artery and tortuous cystic duct may further speed up the process by acting as a rigid fulcrum for torsion [5].

Two types of gallbladder torsion have been described: incomplete (rotation less than 180 degrees), and complete (rotation more than 180 degrees) [3]. Presenting symptoms of gallbladder torsion depend on type of volvulus. Complete torsion presents sudden onset severe right upper quadrant pain, nausea and vomiting, with a tender palpable mass as in our patient. Incomplete torsion presents with recurrent upper abdominal pain with or without vomiting [2,4,6]. In this group, cholelithiasis has been reported in only one quarter of the patients [7].

Preoperative diagnosis of gallbladder torsion is difficult. Clinical presentations are common to other intraabdominal conditions (acute cholecystitis, acute appendicitis). The diagnosis is usually confirmed at laparotomy. The reported findings of gallbladder torsion at US include a floating gallbladder, and thickened gallbladder wall [8]. However, in our case US features reported as an acalculous cholecystitis. The CT features suggesting gallbladder torsion such as horizontal misplaced gallbladder, and fluid collection between the gallbladder and liver bed indicating a 'floating gallbladder' were present in our case. The CT findings of our case were suggested a gangrenous cholecystitis or gallbladder torsion.

## Conclusion

Cholecystectomy is the treatment of gallbladder torsion. Both open and laparoscopic techniques are available for the cholecystectomy. Laparoscopic detorsion and cholecystectomy offers advantages of the minimally invasive surgery [9]. Clinical signs and radiographic studies may be helpful to diagnose of gallbladder torsion. Early diagnosis and surgical treatment lower the mortality of disease. Delayed diagnosis and treatment may give rise to complications.

## Abbreviations

US: Ultrasound; CT: Computed tomography.

## Consent

Written informed consent was obtained from the patient for publication of this case report and accompanying images. A copy of the written consent is available for review by the Editor-in-Chief of this journal.

## Competing interests

I declare that I have no competing interests.

## Authors' contributions

KC performed surgery and writer of manuscript. AP performed surgery and interpreted the patient data. ZK performed and interpreted radiological examinations. TZN reviewer of manuscript.
